# Rheological characterization of an injectable alginate gel system

**DOI:** 10.1186/s12896-015-0147-7

**Published:** 2015-05-06

**Authors:** Benjamin Endré Larsen, Jorunn Bjørnstad, Erik Olai Pettersen, Hanne Hjorth Tønnesen, Jan Egil Melvik

**Affiliations:** School of Pharmacy, University of Oslo, Oslo, Norway; Department of Physics, University of Oslo, Oslo, Norway; FMC Biopolymer AS, Sandvika, Norway; Current address: Elopak AS, Spikkestad, Norway; Current address: Origomar AS, Oslo, Norway

**Keywords:** Alginate gel, Biocompatible, Gelling kinetics, Gel system, Rheological characterization

## Abstract

**Background:**

This work investigates a general method for producing alginate gel matrices using an internal mode of gelation that depends solely on soluble alginate and alginate/gelling ion particles. The method involves the formulation of two-component kits comprised of soluble alginate and insoluble alginate/gelling ion particles. Gelling kinetics, elastic and Young’s moduli were investigated for selected parameters with regard to soluble alginate guluronate content, molecular weight, calcium or strontium gelling ions and alginate gelling ion particle sizes in the range between 25 and 125 micrometers.

**Results:**

By mixing the two components and varying the parameters mentioned above, alginate gel matrices with tailor-made viscoelastic properties and gelling kinetics were obtained. Final gel elasticity depended on alginate type, concentration and gelling ion. The gelling rate could be manipulated, e.g. through selection of the alginate type and molecular weight, particle sizes and the concentration of non-gelling ions.

**Conclusions:**

Formulations of the injectable and moldable alginate system presented have recently been used within specific medical applications and may have potential within regenerative medicine or other fields.

## Background

Hydrogels in the form of cross-linked hydrophilic polymers that adsorb large amounts of water without dissolution are widely used as growth matrices and scaffolds in regenerative medicine, as well as in numerous other biomedical applications [1-6]. Alginates have interesting properties in this respect. They are biopolymers which can be extracted from macroalgae or bacterial cultures and consist of unbranched binary copolymers of 1–4 glycosidically linked α-*L*-guluronic acid (G) and its C-5 epimer β-*D*-mannuronic acid (M) [7].

Both preclinical and clinical studies have shown that alginates are highly biocompatible [8,9]. Because of their ability to form gels under physiologically relevant conditions, they are widely used and studied for encapsulation purposes and as biostructure materials. Entrapment of cells in alginate beads is a commonly used technique [10,11], and alginates have been shown to be a useful and promising material for other types of biostructures. Among other applications, they have been used to bioartificially engineer nerve tissue [12-14], investigate and treat cartilage defects [15,16] and various other tissues [17,18].

Alginate hydrogels are most frequently produced by exposing an aqueous solution of alginate polymer to calcium ions, but many other di- and multivalent metal cations may also be used. As an alternative to Ca^2+^, both Ba^2+^ and Sr^2+^ produce stronger, yet still biocompatible alginate gels [19-22]. Gelling occurs when cations coordinate with the charged substituents of the monomer moieties, leading to the formation of interchain complexes involving several guluronate moieties on adjacent alginate strands. Given sufficient concentration of alginate and binding ions, this gives rise to a three-dimensional network in the form of a gel. The binding zone between the G-blocks is often described by the so-called “egg-box model” [23]. More recent research, however, has shown that other gel formation mechanisms involving alternating (MG) structures also play a significant role [24,25].

Alginate hydrogels are commonly formed by the dialysis/diffusion method, where an alginate solution is gelled by diffusion of gelling ions from an outer reservoir. This method is the most frequently used when making alginate gel beads [22,26]. The manufacturing of alginate microbeads is, however, a rapid process limited by the diffusion of gelling ions into the gel network. It is therefore often less useful in the production of other shapes or structures. This is both because the rapid gelling process limits the time available to mold the gel structure to shape, and because this mode of gelation frequently leads to an inhomogeneous gel [27], as the concentration of alginate in the shaped structure produced will correspond to the distance to the source reservoir, measured along its radius [22].

Alternative methods for manufacturing biocompatible alginate gel structures also exist. The rate of gel formation may be reduced by using internal gelling systems. Here, the gelling ions are released inside the gel as it forms, and the rate of gelation may be controlled by modifying the chemistry of the release. Commonly, a calcium salt with limited solubility, or complexed Ca^2+^ ions, are mixed with an alginate solution. Thereafter, a release of calcium ions is triggered. Calcium sulfate has been used as a cross-linking agent in alginate based cell delivery vehicles for tissue engineering [28-30]. Release of calcium ions and gelling kinetics may also be controlled by using calcium salts with pH-dependent solubility and the addition of a slowly acting acid such as D-glucono-δ-lactone [18,31]. Also, liposomes loaded with calcium ions and designed to rupture under specific conditions have been used in order to form a controllable alginate gelling system [32,33]. Importantly, alginate gel systems based upon internal gelling may have a more defined and limited supply of gelling ions, compared to diffusion systems, where calcium ions are allowed to diffuse into the alginate solution to give a calcium saturated gel.

Alginate gel systems which are formulated with the intent to delay the gelling process could be advantageous for several purposes. In such systems, the gelation could, for instance, be timed or otherwise regulated, to allow the addition of cells or other biomaterials to a carrier liquid which would be sufficiently non-viscous to be injected and thereafter solidify to a firm gel matrix. Several formulations of alginate gel systems may yet have important limitations. Depending on the applications there may be problems associated with the control of the gelling kinetics. Also, systems where gelation takes place under non- physiological pH values have obvious limitations. It may therefore be useful to develop alginate gel formulations with a chemistry that is compatible with a wider range of biomedical applications.

The present method, which is based on a mixture of a suspension of internally cross-linked alginate particles with sodium alginate solution, constitutes such an alternative alginate gelling system. We have here studied several parameters influencing gelling kinetics and elasticity in particular. We have earlier found that the gel system allows conveniently entrapping cells in a 3D environment while preserving a high degree of viability, and that it was useful in a model system for the determination of cell respiration in this respect [34,35]. It has also been useful in constructs for cartilage regeneration and a formulation is currently undergoing clinical trials as a treatment for dilated cardiomyopathy [36-38].

## Results

**Figure 1:**
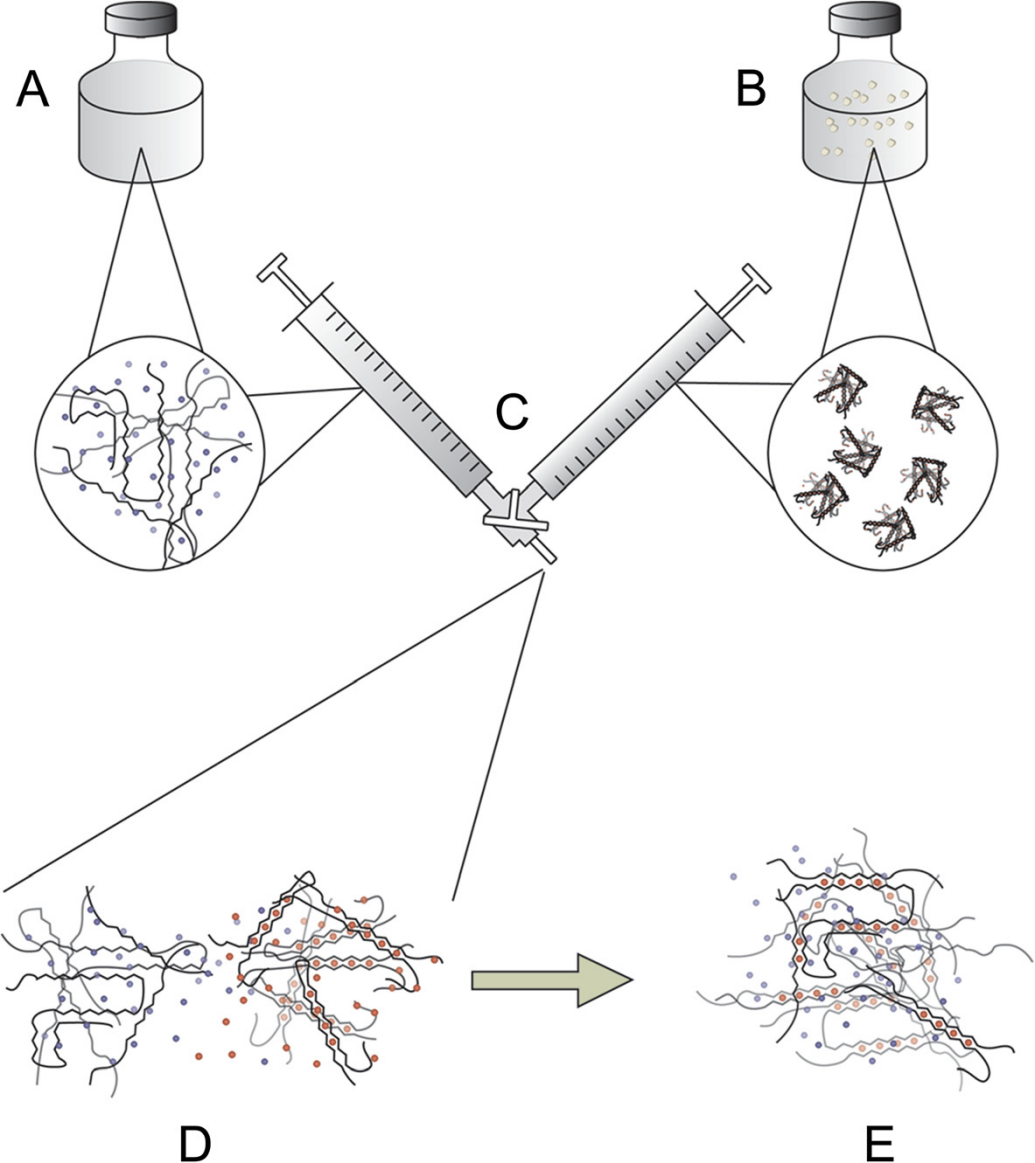
Schematic illustration of the two-component alginate system and the method of gel formation. The system consists of an aqueous sodium alginate solution **(A)**, and insoluble strontium or calcium alginate particles dispersed in an aqueous medium **(B)**. The individual components reside in each of two syringes connected with a three-way connector **(C)**. Upon mixing, gelling ions migrate from the strontium or calcium alginate particles **(D)**. A reciprocal migration of non-gelling ions associated with the soluble alginate also takes place. The gelling ions coordinate with binding sites on two adjacent chains **(E)** and a gel is formed.

**Figure 2:**
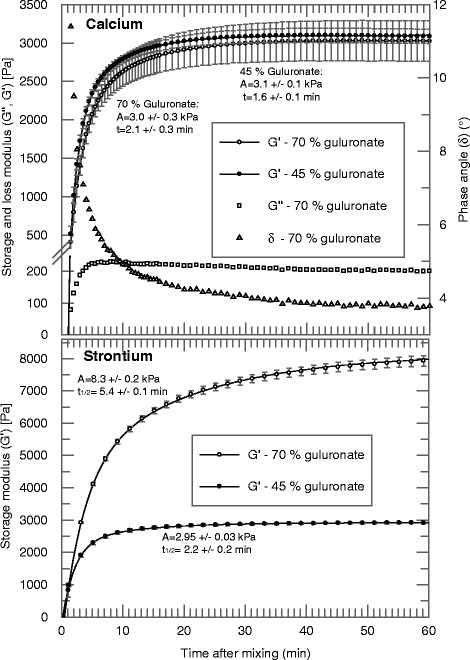
Oscillatory rheological studies of two different sodium alginates. The studies were conducted on two different alginates (*F*_g_ = 0.7 with *M*_*W*_ = 219 kDa, *F*_g_ = 0.44 with *M*_*W*_ = 222 kDa) gelled using calcium (upper) and strontium (lower) alginate particles. All data points are shown as the mean with standard error of at least three independent runs and curves were fitted to the data by eq. 1. Storage (*G’*) and loss (*G”*) moduli, as well as phase angle (tan (*G”*/*G’*)) as a function of time after mixing are shown for gels made of sodium alginate containing ~45% and 70% guluronic acid mixed with calcium alginate. The total alginate salt concentration was 2.0% (w/w) i.e. 1.0% from each of the two components (upper panel), 2.1%, consisting of 1.0% from sodium alginate and 1.1% from strontium alginate (lower panel). Calculated maximum elasticity (*A*) and half time (*t*_1/2_) for the gels are shown.

The gel system studied (Figure 1) allowed variation of several parameters including the guluronate fraction of the soluble alginate, molecular weight and concentrations of the formulation constituents. A detailed description of the alginate components used can be found in Table 1. The alginate particle dispersion could be modified with respect to particle size and gelling ion species. Mixing the components using the two connected syringes took less than 5 seconds, and after the viscous gelling mass was ejected onto the serrated holding plate of the rheometer the first measurement could be recorded within about 90 seconds. In Figure 2B the development of storage moduli over time is shown for high-guluronate strontium alginate particle dispersions in combination with two different sodium alginate solutions. The sodium alginates had similar molecular weight, but differed in their composition with respect to the guluronic to mannuronic acid ratio. Formulations made using the same sodium alginates and a dispersion of high-mannuronate calcium alginate particles were also investigated (Figure 2A). Storage modulus, *G’*, as a function of time is shown for both alginates, while loss modulus, *G”*, and phase angle *i.e.* arctan(*G”*/*G’*), are shown only for the high guluronate alginate, in order to increase the legibility of the figure as it showed a similar tendency.

**Table 1:** **Alginate gelling formulations teste** **d**

**Figure**	**Sodium alginate**			**Ca/Sr alginate**			
	**Concentration**	**F**_**G**_	**M**_**W**_	**Concentration**	**F**_**G**_	**Bound ion**	**M**_**W**_
Figure 2 (lower panel)	1.0%	0.70	219 kDa	1.1%	0.65	Sr	130 kDA
	1.0%	0.44	222 kDa	1.1%	0.65	Sr	130 kDA
Figure 2 (upper panel)	1.0%	0.70	220 kDa	1.0%	0.47	Ca	176 kDa
	1.0%	0.45	222 kDa	1.0%	0.47	Ca	176 kDa
Figure 3	1.0%	0.69	257 kDa	1.1%	0.65	Sr	130 kDA
	1.0%	0.69	70 kDa	1.1%	0.65	Sr	130 kDA
	2.0%	0.69	70 kDa	1.1%	0.65	Sr	130 kDA
Figure 4	1.0%	0.69	219 kDa	1.1%	0.65	Sr	130 kDA
Figure 5	0.4-1.6%	0.69	219 kDa	0.4-1.6%	0.65	Sr	130 kDA
	0.4-1.6%	0.69	219 kDa	0.4-1.6%	0.47	Ca	176 kDa
Figure 6	1.0%	0.69	219 kDa	1.1%	0.65	Sr	130 kDA
Table 2	1.0%	0.45	220 kDa	1.0%	0.47	Ca	176 kDa
	1.0%	0.70	222 kDa	1.0%	0.47	Ca	176 kDa

The given concentrations are for the final gel mixture. F_G_ is the guluronate fraction. M_W_ is the molecular weight.

Gel formation occurred rapidly during the first minutes and the storage modulus subsequently showed a stabilizing tendency as a result of the sol–gel transition. A high content of guluronic acid resulted in a storage modulus that was almost three times greater than for the formulation with lower guluronic acid content in the case of gels formed using strontium alginate dispersions (Figure 2B). The gel formed using high-guluronate sodium alginate gelled with high-mannuronate calcium alginate dispersion had similar characteristics as that of the gel made using high-mannuronate sodium alginate gelled with high-guluronate strontium alginate. These formulations were also subjected to compression tests (Table 2), where analyses showed that gels formed in the absence of exogenous substances had similar elastic properties, as measured by Young’s modulus, although they differed with respect to maximal compressibility, as measured by true strain at maximal corrected stress.

**Table 2:** **Compression tests on gels with different guluronate content with or without treatment with additional calcium**

	**~45% guluronate, no treatment**	**~70% guluronate, no treatment**	**~45% guluronate, Ca-treated**	**~70% guluronate, Ca-treated**
Number of tests (n)	6	8	4	6
Young’s modulus (*E*, kPa)	20 ± 3	21 ± 1	90 ± 10	120 ± 10
Maximal stress (*σ*_*c,u*_, kPa)	6.4 ± 0.2	9.8 ± 0.4	59.0 ± 0.8	85 ± 4
Hencky strain at maximal stress (*ε*_*h,u*_)	0.39 ± 0.01	0.62 ± 0.01	1.08 ± 0.04	0.92 ± 0.09

Compression tests were performed on gels made from 1.5 ml 2% sodium alginate solution with a guluronate content of ~45% or ~70% and 1.5 ml of 2% calcium alginate dispersion. Gels were either left in the mold for approx. 3 hrs or left in the mold for 1.5 hrs and subsequently incubated in 0.55% w/w CaCl_2_ and 0.61% w/w NaCl for 2 hrs. Engineering stress (*σ*_*eng*_) and strain (*ε*_*eng*_) values were converted to corrected stress (*σ*_*c*_) and Hencky’s strain (*ε*_*h*_) according to eqs. 2 and 3. Young’s modulus was calculated as the initial linear slope of the resultant stress–strain plot. Ultimate stress was calculated as the upper inflection point of the stress–strain curve. In the case of Ca^2+^-treated gels, the inflection point, rather than an abrupt break, was the top of a plateau. All values are given as mean ± standard error, with the number of parallels given in the first row. There is no statistically significant difference between the Young’s moduli of gels having received the same treatment. Gels of similar composition (containing ~45% and ~70% guluronate in the sodium alginate component) but treated differently, show a statistically significant difference at the p < 0.01 and p < 0.001 level (based on corrected readings). For each formulation and treatment, maximal stress was statistically different from the others at p < 0.005. For untreated gels, the strain at maximal stress was statistically different at the p < 10^−7^ level for both corrected and uncorrected measurements. There was no such difference for treated gels (p = 0.37 and p = 0.15, respectively).

**Figure 3:**
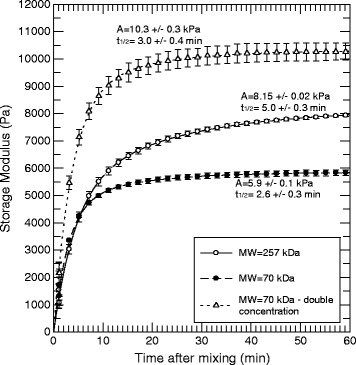
Storage modulus and kinetics of alginate gels as a function of time and molecular weight. Guluronate-rich sodium alginates (*F*_g_ = 0.7) with different *M*_*W*_ were combined with strontium alginate. The total alginate salt concentration was 2.1%, consisting of 1.0% from sodium alginate and 1.1% from strontium alginate except for the upper curve where the sodium alginate concentration was increased to 2.0% (total alginate concentration was 3.1%). All data points are shown as the mean with standard error of three independent runs and curves were fitted to the data by eq. 1. Calculated maximum gel strength (*A*) and half time (*t*_*1/2*_) for the gel setting are shown.

A further experiment was performed in order to study the influence of the molecular weight and alginate concentration on the gel formation. An alginate batch with a high content of guluronic acid was degraded by autoclaving the alginate solution [39] from a molecular weight of 257 kDa to 70 kDa and used in the gel formulation. The data shown in Figure 3 demonstrate both a higher gelling rate for the light alginate and that the gel stabilized at lower elasticity values. Increasing the soluble alginate concentration in the formulation from 1.0% to 2.0% resulted in an approximately two-fold increase in the storage modulus, but did not significantly change the gelling rate (3.0 vs. 2.6 minutes).

**Figure 4:**
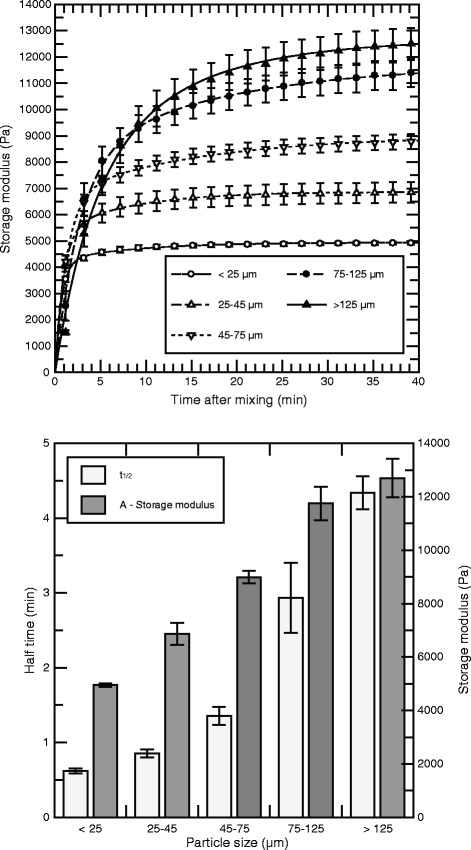
Storage modulus and kinetics of alginate gels as a function of particle size. Upper panel: Storage modulus of alginate gels as a function of time for gels made of sodium alginate (*F*_g_ = 0.7 and *M*_*W*_ = 219 kDa) and Sr alginate at different particle sizes. The total alginate concentration was 2.1%, consisting of 1.0% from sodium alginate and 1.1% from Sr alginate. The curves were fitted by eq. 1 to the average data from three independent runs. Lower panel: Calculated maximum storage modulus (*A*) and half time (*t*_*1/2*_) for the fitted data. Error bars denote the standard error of the mean calculated at each data point, and are shown when exceeding the dimension of point markers.

The effect of different particle size distributions was studied by subjecting the same calcium alginate batch to milling and sifting using a range of sieves. The self-gel formulations were gelled with the same solution of sodium alginate (Figure 4). Although the concentrations of both alginate components in the formulations tested were always equal, the properties of the resulting gels were quite different: Smaller particles led to a higher rate of gelation and a lower final storage modulus, whereas larger particle size resulted in lower gelling rates and higher gel elasticity. For the smallest particle sizes (less than 25 μm) it should be noted that, because of the high gelling rate, some mechanical degradation of the gel probably occurred while the two components were still being displaced between the syringes.

**Figure 5:**
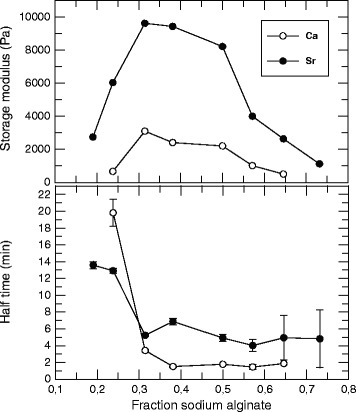
Storage modulus of alginate gels as a function of alginate fraction in solution or particulate. Final storage modulus (*A*) and half time (*t*_*1/2*_) calculated from fitted oscillatory rheology curves, as a function of the fraction of sodium alginate (*F*_g_ = 0.7 and *M*_*W*_ = 219 kDa) in a mixture with Sr alginate or Ca alginate. The total alginate concentration was always 2.0%. Bars demonstrating standard error of the mean are indicated when exceeding the symbols.

The relative contribution to gelation half time and final storage modulus of the sodium alginate solution and gelling ion-alginate dispersion was also further explored (Figure 5): A series of oscillation experiments were performed where the relative content of the components were varied while keeping the total alginate concentration constant at 2.0%. All shear stress-time curves obtained from each formulation (not shown) were fitted by non-linear regression as described above, and the half time and final gel elasticity were plotted against the fraction of sodium alginate in the formulations. The curves were obtained both for calcium and strontium alginate particles of the same sizes (45–75 μm). The final gel elasticity as well as gelling rate seemed to follow a similar pattern for both types of particles although the final storage modulus was several times higher and gelation half time generally shorter when using strontium alginate as the gelling ion donor. At the concentrations tested, maximum gel elasticity values were obtained when the soluble alginate fraction was kept at about 30% of the total alginate content. Increasing the fraction of sodium alginate above this level resulted in reduced gel elasticity, obviously as a result of a reduction in the gelling ion concentration. For these observations, however, the gelling rate seemed to remain constant, independently of the alginate concentration. Decreasing the sodium alginate concentration below 30% also resulted in a lower final storage modulus, but was in this case followed by a reduction in the gel formation rate.

**Figure 6:**
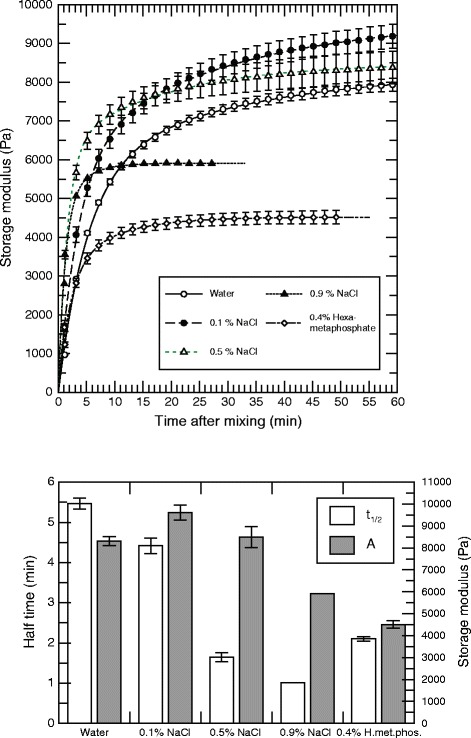
Storage modulus and kinetics of alginate gels as a function of non-gelling salts. Upper panel: Storage modulus of alginate gels as a function of time for gels made of sodium alginate (*F*_g_ = 0.7 and *M*_*W*_ = 219 kDa) and Sr alginate in the presence or absence of sodium chloride or hexametaphosphate. The total alginate concentration was 2.1%, consisting of 1.0% from sodium alginate and 1.1% from Sr alginate. Oscillatory stress-time curves were fitted to the data as described to obtain final storage modulus (*A*) and half time (*t*_*1/2*_). Lower panel: Final storage modulus and half time (with standard error), calculated from fitted curves. All data points are shown as the mean with standard error of three independent runs (only one run for 0.9% NaCl).

The gelling process was found to be strongly influenced by the presence of non-gelling ions. In Figure 6, a series of oscillatory shear stress measurements performed on formulations with different concentrations of sodium chloride is shown. The gelling rate increased strongly in the presence of sodium ions. For concentrations up to 0.5% NaCl the final gel elasticity was found to be relatively constant. The reduced gel elasticity observed at 0.9% NaCl was likely due to the fact that the gel was partly disrupted due to rapid gelling before the gel/solution was placed under the rheometer probe, similarly to the formulations having the smallest particle size. Not surprisingly, the final gel strength was lowered in the presence of a strontium binding compound, hexametaphosphate, and this was also found to increase the gelling rate. The data shown in Figure 6 were obtained from gels made using strontium alginate as the gelling ion source. A similar dependency was also found when using calcium alginate as gelling ion source (data not shown).

## Discussion

The new alginate gelling system developed here is based upon the exchange of gelling ions between soluble and insoluble alginate fractions. Gel formation was demonstrated by the elasticity build up and the phase angle and loss modulus time series confirmed the rapid sol–gel phase transition (Figure 2A). The sol–gel transition of alginates is regulated by the availability of free gelling ions and has previously been studied in details [40-43]. The low phase angle observed in our experiments indicated that the sol–gel transition occurred already before the start of the measurements. In contrast to dialysis gelling from an external reservoir commonly used, which will saturate the gel with gelling ions [44], the current *in situ* gel formulations is limited with respect to availability of gel forming ions like Ca^2+^ and Sr^2+^ which was tested here. The limitation in gelling ions will have effects on the gel system which is clearly demonstrated by the change in strength and elasticity by adding extra gelling ions (Table 2). It is well known that alginates with a higher content of guluronic acid may give higher gel strength, but this may not be the case at lower gelling ion concentrations [25,45]. For calcium and strontium based gels there also seemed to be a difference in gelling kinetics and elastic behavior between the two alginate types with a large difference for the strontium based gels (Figure 2B). The effects may be due to differences in flexibility between the two alginate types and the difference in gel forming properties between calcium and strontium [24] The lack of difference in elasticity when using calcium alginate particles was also demonstrated by the calculated Young’s modulus, although the data differed significantly with respect to compressive strength (Table 2).

Higher molecular weight alginate chains resulted in a higher elastic modulus in the gels that were formed, as did an increase in alginate concentration (Figure 3). However, while more than tripling the molecular weight of the sodium alginate component led to an increase in elastic modulus with a concomitant increase in gelling half time, doubling its concentration nearly doubled the elastic modulus with no significant change in the gelling kinetic. The *M*_*W*_ dependency may be interpreted as the result of faster availability of shorter chains during gelling while the formed gel may suffer with less entanglement or *M*_*W*_ overlap. A reduced gelling rate may therefore be obtained by using high *M*_*W*_ alginates. However, for applications using the current gel system for cell administration, the use of higher concentrations of high *M*_*W*_ alginates in particular may be limited by the high viscosity [46]. Although there are no established limits for the viscosity of injectable preparations [47], very high (*i.e.* > 3.0%) concentrations of sodium alginate might yield solutions that are too viscous to be practically useful [48]. It has also been demonstrated that certain ranges of elasticity in hydrogel cell carriers may have a cytoprotective effect [49]. The formulations described here clearly have sufficiently low viscosity during the first minutes after mixing the two components to allow administration through commonly used syringes and to be used in combination with cells.

Increasing the particle size of the calcium alginate component (Figure 4) led to gelation responses which were mainly a function of the calcium release kinetics: Smaller particles will, due to the increase in surface area per total particle weight, lead to a higher rate of calcium ion release into the gel-forming mix. This in turn leads to a lower gelation half time and lower elastic modulus in the final gel. The latter, it can be speculated, is the result of rapidly setting gels being stabilized at non-optimal thermodynamic minima, while longer gelation times allow for the evolution of more optimal cross-linking configurations.

The overall effect of varying the relative amount of the two components on the storage modulus appears similar for both gelling ions (Figure 5), and was found to be in the optimal range with respect to the magnitude of *G’* and gelling half time when the sodium alginate fraction was about 30%. The difference between the two gelling ions was significantly stronger for guluronate-rich gels, a result that is in agreement with previous works by Mørch *et al.* [22], and may be a result of differences in the mode of complex formation with the uronic acid residues on the alginate chains [24,25]. The data also demonstrated that the use of calcium alginate particles gave a higher gelling rate compared to similar strontium alginate particles (Figure 5), which may reflect the difference in binding mechanisms. However, when the sodium alginate concentration was reduced below about 30% of the total alginate content of a formulation (amounting to 2% w/w of the aqueous two-component system), gelling half time was increased for both particle types (Figure 5). As the particles were found to remain insoluble and stable for several months in aqueous solutions (data not shown) this might be the result of approaching a threshold level for the effect of the soluble alginate concentration.

The rate of gelation was strongly increased by the addition of sodium ions to the sodium alginate solution (Figure 6). The presence of an excess of sodium ions in the gelling mix increases the rate of ion exchange with the calcium donor due to entropic forcing. At sufficiently high concentrations of sodium chloride this might have led to gel stabilization at more entropic thermodynamic minima, similar to what was previously discussed for smaller calcium alginate particles (Figure 4). The results demonstrated that an isotonic sodium chloride concentration of 0.9% might result in a formulation which may not be useful for injection, as passage through a syringe will partly destroy the gel network. This has been seen in other two-component systems as well [50], and may be avoided by using a secondary, neutral tonicity regulator such as mannitol or glucose.

The addition of a calcium binding agent, sodium hexametaphosphate, resulted in a marked reduction in final storage modulus due to the overall constraint in the availability of calcium (Figure 6). The amount added was sufficient to bind a large portion of the total amount of calcium present in the system. The observed increase in the rate of gelation strengthens this view, as thermodynamic equilibrium would be obtained more rapidly in a calcium-limited gel, because of a reduction in the number of possible coordination sites.

Our interpretation of the data presented here is that the most important factor influencing the gelling rate was the rate of gelling ion release from the particles. This is supported by the very quick gelling that occurs in contact with alginate and free gelling ions. The two-component kinetic equation used could be well fitted to all measured elasticity data (although not for wider particle distributions, results not shown). A first-order equation could not be used to fit our data, but has previously been found useful for glucono-delta-lactone initiated release of Ca^2+^ ions from CaCO_3_ [45]. It can be speculated that the dual kinetic behavior may result from ion release taking place both from the particle surface – an effect that is related to particle size – and from the particle interior, as particles unfold.

The results of the measurements of storage modulus as a function of time correspond well with previously published work on the elasticity of alginate gels [51,52]. Furthermore, the effects of an increase in the number of cross-links were similar to those in gels produced using a concentration-dependent internal mode of gelation based on hydrophobic association of modified alginate amides [53]. Variations in structure elasticity which has been shown to influence differentiation and function of cells in 3D cultures [54,55], is – within a certain range – possible to regulate by varying the formulation parameters.

Due to the high biocompatibility of alginates an advantage with the present method compared to many other alternatives may be that alginate and gelling ions are the only materials used, thus the chemical complexity is reduced. As mentioned earlier the gel technology presented here is currently being tested in preclinical and clinical models. This together with the tunable properties of the gel system demonstrated here therefore indicates potential other uses, like within cell and biofactor administration, cell cultivation, tissue bulking and tissue engineering.

## Conclusion

In the present work, a system that allows the formulation of injectable alginate gels with an internal mode of gelation was presented. The effects of calcium and strontium as gelling ions, the ratio of guluronate to mannuronate in the alginate, its molecular weight, alginate concentration and particle distribution were shown to influence the storage modulus and gelling kinetics. The two-component alginate system presented produces injectable and moldable gel matrices of a material that several studies have shown to be a biocompatible and versatile matrix for use in regenerative medicine and other biomedical applications.

## Methods

### Alginates

All samples tested were made from PRONOVA sodium alginates (FMC Biopolymers/NovaMatrix, Norway). Insoluble calcium and strontium alginates were prepared from sodium alginate by precipitation of alginate solutions (1–2%) with calcium or strontium salts solutions before freeze-drying and milling. The gels were mechanically fragmented and washed repeatedly in water to remove excess Ca or Sr ions before drying and the final product was measured to contain approximately stoichiometric amounts of Ca (~10%) or Sr (~20%). Final particle size was controlled by an adapted milling and sieving procedure. The molecular weight of alginate samples was determined by size-exclusion chromatography with light scattering detection (given as weight average) and the sequence distribution of guluronic and mannuronic acid was determined by NMR spectroscopy. All strontium alginate samples were of high guluronic acid content (~70%). Details on the alginates used in each experiment can be found in Table 1.

### Gel preparations

Solutions of sodium alginates at appropriate concentrations were made by adding a corresponding amount of solvent to vials containing a standardized amount of freeze-dried alginate and stirring or shaking the vials for at least one hour. This resulted in clear and viscous solutions. Dispersions of calcium and strontium alginates were prepared before use as the alginate particles was insoluble in water in the absence of soluble alginate. The solution of soluble alginate and calcium or strontium alginate dispersion was conveniently mixed by using two syringes connected with a Luer lock connection and using a manifold (Connecta Plus 3, Becton Dickinson Infusion Therapy AB). The contents of the two syringes were mixed by first displacing the sodium alginate solution into the syringe containing the strontium or calcium alginate dispersion; the resultant mixture was displaced back and forth between the syringes nine times before the gelling mix was emptied through the manifold’s third channel into an appropriate container and allowed to form a gel (Figure 1). The displacement itself was performed in a rehearsed manner and timed to a maximum of 5 seconds. For sterile gel preparations 0.22 μm filtered alginate solutions and insoluble alginate dispersions sterilized by autoclaving were used.

### Oscillatory rheological measurements

The setting of the gel with time (storage modulus) was measured using a Physica MCR 300 rheometer (Measuring system: PP50, serrated probe, gap: 1 mm, frequency: 1 Hz and strain: 0.005). Gels were prepared as described above and deposited directly onto the serrated base of the rheometer. The probe was subsequently lowered and low-viscosity silicone oil was applied at the outer edges in order to prevent evaporation from the gel. The set-up of the experiments was timed to 60 seconds from the first syringe-to-syringe displacement.

The strain setting was chosen based on strain sweep experiments determining the viscoelastic window of the gels under investigation (results not shown), and the temperature was kept at 20°C during the experiments. In all cases it was empirically found that the rheological data could be well fitted to equation (1),

1 $$ G\hbox{'}=A\left(1-f\cdot {e}^{-{k}_1t}-\left(1-f\right){e}^{-{k}_2t}\right) $$

where *G’* is storage modulus, *t* is time, *A* is final gel strength, *k*_*1*_ and *k*_*2*_ are rate constants and *f* is a relative contribution fraction between the two exponential parts. Curve fittings and drawings were performed by using GraFit 5.0 (Erithacus Software, Horley, UK), except for the curves in Figure 3, which were drawn in Origin 8.0 (OriginLab, Northampton, US-MA).

### Compression tests

Fully set gels were examined using a TA.XT2 Texture Analyzer (Texture Technologies Corp., Scarsdale, NY/Stable Micro Systems, Godalming, Surrey, UK). After mixing the gel components between syringes, the gel mass was transferred to 24-well plates, which were used as molds, and left to set for 1.5 hrs. After this period, some samples were carefully extracted from the wells using a flat spatula and cut to 5 mm thick slices, which were transferred to 6-well culture plates containing an aqueous solution with 0.55% w/w CaCl_2_ (50 mM) and 0.61% w/w NaCl and left to incubate for 3 hrs. The remaining samples were kept in the mold for the same duration of time, and were subsequently extracted in the same manner and cut to 5 mm thick slices.

Excess saline solution was carefully wiped off and the specimens were transferred to the Texture Analyzer’s sample container. An S/N4 cylindrical flat-tip probe with a cross-section of 4 mm was used. The instrument was programmed to measure force while the probe traversed the 6 mm gap between the probe’s initial position and the sample container surface at a speed of 3 mm/min. Raw data were imported to MATLAB® R2010a (The Mathworks, Natick, MA). A custom script was used to remove head space readings, convert position data to engineering strain and force readings to pressure. The data were in turn converted to Hencky’s (true) strain and corrected stress using equations (2) and (3), according to the procedure used by Mao et al. [39]. Young’s modulus was found as the slope of a linear regression over the data part recorded at the engineering strain interval of 0 – 0.05, i.e. up to 5% compression.

2 $$ {\varepsilon}_h=- \ln \left(1-{\varepsilon}_{eng}\right) $$

3 $$ {\sigma}_c={\sigma}_{eng}\left(1-{\varepsilon}_{eng}\right) $$

### Statistical analyses and data processing

All statistical analyses were performed in Excel 2010 (Microsoft, Redmond, US-WA), using at least three parallel measurements. The threshold of statistical significance was set at *p* < 0.05, unless otherwise detailed.
